# A Comprehensive Excursus of the Roles of Echocardiography in Heart Transplantation Follow-Up

**DOI:** 10.3390/jcm13113205

**Published:** 2024-05-29

**Authors:** Daniela Bacich, Chiara Tessari, Giulia Ciccarelli, Giovanni Lucertini, Alessia Cerutti, Nicola Pradegan, Giuseppe Toscano, Giovanni Di Salvo, Antonio Gambino, Gino Gerosa

**Affiliations:** 1 Cardiac Surgery Unit, Department of Cardio-Thoracic-Vascular Sciences and Public Health, University Hospital of Padova, 35128 Padova, Italy; daniela.bacich@aopd.veneto.it (D.B.); giulia.ciccarelli@studenti.unipd.it (G.C.); giovanni.lucertini@studenti.unipd.it (G.L.); nicola.pradegan@unipd.it (N.P.); giuseppe.toscano@aopd.veneto.it (G.T.); antonio.gambino@unipd.it (A.G.); gino.gerosa@unipd.it (G.G.); 2 Pediatric Cardiology Unit, Department of Women’s and Children’s Health, University Hospital of Padova, 35128 Padova, Italy; alessia.cerutti@aopd.veneto.it (A.C.); giovanni.disalvo@unipd.it (G.D.S.)

**Keywords:** heart transplantation, echocardiography, coronary flow reserve, Doppler tissue imaging, strain rate imaging, stress imaging

## Abstract

Current guidelines for the care of heart transplantation recipients recommend routine endomyocardial biopsy and invasive coronary angiography as the cornerstones in the surveillance for acute rejection (AR) and coronary allograft vasculopathy (CAV). Non-invasive tools, including coronary computed tomography angiography and cardiac magnetic resonance, have been introduced into guidelines without roles of their own as gold standards. These techniques also carry the risk of contrast-related kidney injury. There is a need to explore non-invasive approaches providing valuable information while minimizing risks and allowing their application independently of patient comorbidities. Echocardiographic examination can be performed at bedside, serially repeated, and does not carry the burden of contrast-related kidney injury and procedure-related risk. It provides comprehensive assessment of cardiac morphology and function. Advanced echocardiography techniques, including Doppler tissue imaging and strain imaging, may be sensitive tools for the detection of minor myocardial dysfunction, thus providing insight into early detection of AR and CAV. Stress echocardiography may offer a valuable tool in the detection of CAV, while the assessment of coronary flow reserve can unravel coronary microvascular impairment and add prognostic value to conventional stress echocardiography. The review highlights the role of Doppler echocardiography in heart transplantation follow-up, weighting advantages and limitations of the different techniques.

## 1. Introduction

Heart transplantation (HT) is the gold-standard therapy in advanced heart failure, providing survival gain and health-related quality of life [1]. However, HT patients remain at risk of developing complications during follow-up, including acute graft rejection (AR), coronary allograft vasculopathy (CAV), infections, cancer, and renal failure. Timely diagnosis of AR and CAV is mandatory for graft and patient survival. Current guidelines recommend routine invasive screening with endomyocardial biopsy (EMB) and invasive coronary angiography (ICA) at preset intervals as the gold standards for their early detection [2]. While carrying the risk of procedural complications, EMB may provide false negative histological results, due to the patchy nature of AR. Furthermore, EMB in infants and children is a problematic gold standard, and some centers seek to reduce the incidence of its use, limiting it in case of “clinical suspicion”. ISHLT guidelines for the care of pediatric heart transplant recipients suggest using detailed echocardiographic assessments [2]. ICA gives insight into the anatomy of major coronary vessels, but it lacks sensitivity in detecting diffuse concentric lesions, like those seen in early CAV, as it cannot visualize beyond the arterial lumen, and is not capable of wall and lesion characterization. Therefore, it needs to be performed in combination with intravascular ultrasound (IVUS) or optical coherence tomography (OCT) [3]. Furthermore, ICA only gives direct information about epicardial vessels, not about microcirculation. In order to avert the potential procedural risks of intracoronary diagnostics, non-invasive modalities, like coronary computed tomography angiography (CCTA) and cardiac magnetic resonance, have emerged in the long-term follow-up of HT patients [4]. Due to the high prevalence of renal impairment in HT recipients, these techniques still carry the risk of contrast-related acute kidney injury (AKI) and may not be adopted in the long-term follow-up in all patients [5].

Transthoracic echocardiography (TTE) is the first-line imaging modality for the investigation of HT recipients. It can be performed at bedside, serially repeated, and does not carry the burden of contrast-related AKI and procedure-related risk. It provides assessment of left and right ventricular systolic and diastolic function, valvular heart disease, pulmonary hypertension, and pericardial effusion. The additional introduction of Doppler tissue imaging (DTI), speckle tracking echocardiography (STE), stress echocardiography and coronary flow reserve may play a role in the early detection of AR and CAV.

In this article, we review the basic principles of echocardiography in HT recipients, weighing the advantages and limitations of the different techniques.

## 2. Echocardiography of the Normal Cardiac Allograft

Echocardiographic evaluation of HT patients is based on the assessment of the same parameters used for the general population. However, orthotopic HT involves many factors that affect myocardial function, such as abnormalities of the donor heart, physiologic changes of the allograft and pathologic changes occurring during the peri- and post-transplant period [6]. Therefore, echocardiographic assessment of the cardiac graft is complicated by the greater variability of echocardiographic parameters in this cohort compared with the general population and by a lack of specific reference values for this population [7]. Instead of relying on the absolute value of each measurement, it becomes of paramount importance to obtain a baseline evaluation, with which later results should be compared, in order to detect pathological changes over time. This comprehensive evaluation should be performed at least 6 months after HT [8].

### 2.1. Evaluation of Left Ventricular Function

Rejection-free grafts with normal coronary arteries exhibit diastolic left ventricular (LV) volumes in the lower normal range, mild LV hypertrophy and increased LV mass compared with reference values [6,7]. In the first post-transplant period, the increase in LV wall thickness may be explained with peri-transplantation injury and graft edema. In the long term, the persistence of LV hypertrophy is multifactorial: possible mechanisms include hypertension, the effect of immunosuppressive treatment (especially calcineurin inhibitors and prednisone) and hypertrophy of the implanted heart; still, a progressive increase in ventricular wall thickness may be associated with acute graft rejection [9].

Mean LV ejection fraction (EF) is within the normal range. However, like in other forms of cardiac surgery, patients frequently exhibit abnormalities of interventricular septum motion and thickening [6]. Although normal EF does not exclude significant AR or CAV, a progressive decrease in LV systolic function may be a consequence of both AR and CAV in the first post-transplant year. Late reduction of LV ejection fraction is frequently due to CAV progression and carries a poor prognosis [10].

### 2.2. Evaluation of Right Ventricular Function

Both transversal diameter measurements and area measurements of the right ventricle (RV) are increased compared with normal reference values, while conventional parameters measuring RV function, such as tricuspid annular plane systolic excursion (TAPSE), S’ wave at tissue Doppler imaging, fractional area shortening, and global longitudinal strain of the RV free wall, are lower compared with normal [7]. In the early post-transplant period, the dilatation is mainly due to afterload mismatch with relatively high pulmonary pressures of the recipient and loss of pericardial constraint [11,12]. Normalization of RV cavity sizes usually occurs within a few weeks, along with the progressive decrease in pulmonary vascular resistances. Conventional parameters of RV longitudinal function only partially recover within the first year. This can be explained by many factors, like prolonged ischemia time, tricuspid regurgitation, pre-transplant pulmonary pressures, cause of the donor death, and status of the donor heart [13]. Being unable to discern active contraction from passive entrainment caused by the left ventricle, TAPSE and S’ are not to be considered sensitive parameters of global RV function after cardiac surgery. On the other hand, 2D STE echocardiographic assessment of RV longitudinal strain is less angle- and load-dependent and less confounded by RV geometry. RV longitudinal strain is decreased even in healthy HT recipients (−16.9 ± 4.2%) when compared with reference values for the general population [7]. This reflects the changes in RV contractile pattern cardiac surgery, with a relative loss of longitudinal shortening and gain in transverse shortening even in the case of preserved global RV function [14]. Nonetheless, RV global and free wall longitudinal strain are decreased in the early postoperative period and gradually improve within the first year [15,16]. Therefore, a reduction in these parameters over time must be regarded as a pathological finding and prompt further evaluation of possible causes, including rejection but also cardiac allograft vasculopathy, hypertension or infection. Since RV global longitudinal strain includes strain of the right side of the inferior septum, which can be affected by EMB-related myocardial fibrosis, RV free wall strain is likely a more objective parameter of RV function in patients undergoing multiple endomyocardial biopsies [17]. Three-dimensional (3D) echocardiography overcomes the limitations of 2D imaging by assessing RV volume and function without any geometrical assumption [18] and can provide measurements that favorably compare with cardiac magnetic resonance data [19]. For these reasons, newer techniques like STE and 3D echocardiography may be useful tools when assessing RV function in this patient group.

### 2.3. Evaluation of Postoperative Valvular Function

**Figure 1:**
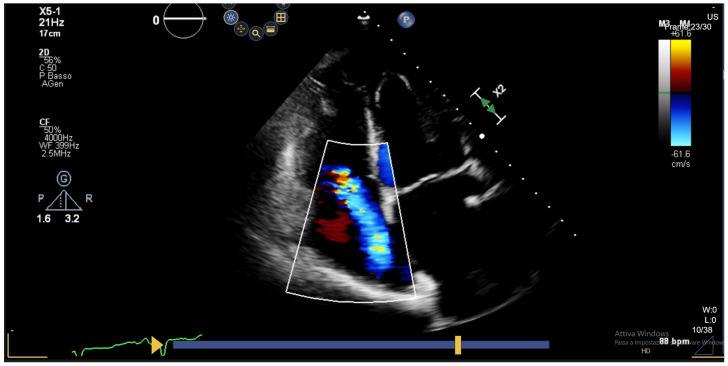
Color Doppler assessment of a severe functional tricuspid regurgitation due to annulus dilatation.

Tricuspid regurgitation (TR) is the most common valvular abnormality in transplanted hearts, with a reported incidence ranging between 19 and 84%. The variability depends on differences in the definition of significant regurgitation and the surgical technique adopted for HT [20]. In the first post-transplant weeks, TR can be attributed to pulmonary hypertension. Its severity decreases spontaneously as pulmonary resistance decreases. The main causes of functional TR beyond the early postoperative period are persistent high pulmonary pressures, enlargement of the tricuspid annulus induced by right ventricular dilatation (Figure 1), and geometric distortion of the tricuspid annulus, the latter being more frequent in the case of the biatrial anastomosis implantation technique. In fact, the adoption of the bicaval technique has significantly reduced the prevalence of early tricuspid regurgitation in HT recipients [21]. Organic tricuspid regurgitation is mainly due to lesions of the chordal apparatus caused by repeated EMBs, with strong correlation between the number of performed biopsies and the severity of TR [22].

Mild mitral regurgitation, due to papillary muscle edema, is common in the early post-transplant period and usually decreases over time [10,23]. Doppler flow velocities at aortic and pulmonary level are usually normal. On some occasions, the mismatch in the size of the donor and recipient pulmonary artery and the suture line in the proximal pulmonary artery may result in an aspect of “pseudo-narrowing”. However, the detection of significant gradients by Doppler is uncommon [8].

### 2.4. Evaluation of Atrial Function

**Figure 2:**
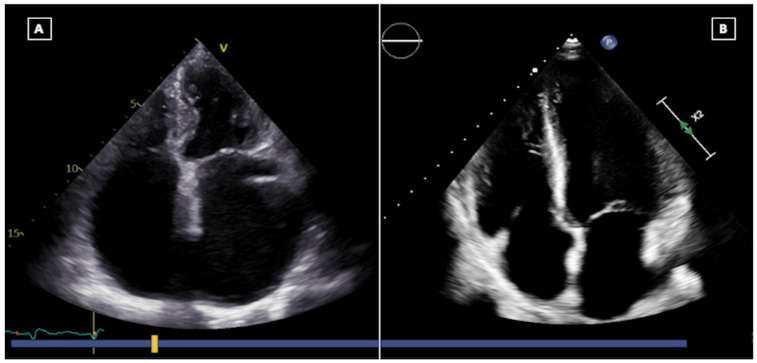
Four-chamber view of a heart transplantation performed through biatrial technique, showing how the atrial chambers are enlarged (**A**); and through bicaval technique, showing how atrial geometry is better preserved (**B**).

Atrial morphology is widely affected by surgical technique. The biatrial surgical approach resulted in bilateral enlargement of the long axis dimension of the atria, with an echo-dense ridge at mid-atrial level, being the site of the anastomosis, best seen in the apical 4-chamber view [24] (Figure 2A). The ridge resulted in impaired atrial hemodynamics, with abnormal LV filling pattern, valve annulus distortion and blood stasis within dilated atria [25]. With the now more widely adopted bicaval technique, atrial geometry is better preserved (Figure 2B). Nevertheless, patients may exhibit significant atrial enlargement, mainly correlated with allograft age [7].

### 2.5. Evaluation of Diastolic Function

**Figure 3:**
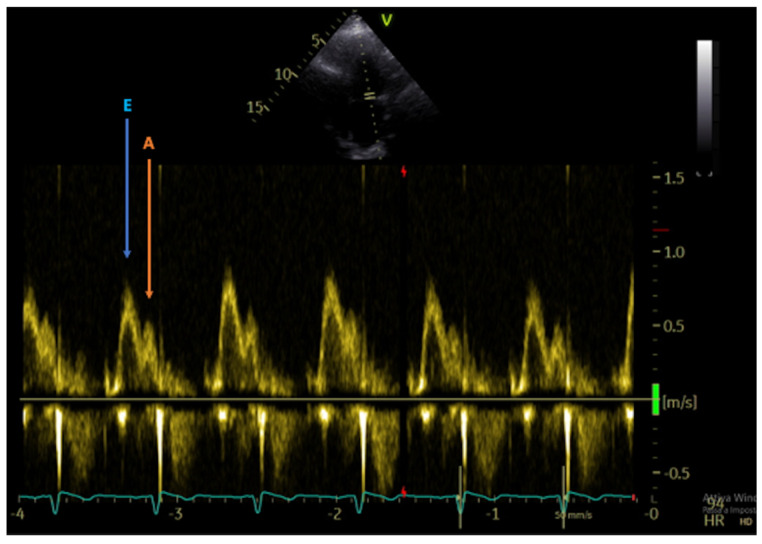
Doppler image showing the overlap of E wave (blue arrow) and A wave (orange arrow) due to the elevated heart rate (HR 94 bpm).

Evaluation of diastolic function after HT using conventional Doppler technique is challenging: the elevated heart rate usually seen in the denervated heart results in overlapping of E and A waves (Figure 3). Moreover, the assessment of LV filling is influenced by many factors, including preload conditions, atrial dynamics and morphology, LV compliance and contractility, end systolic volume and heart rate. In fact, even in the absence of AR or CAV, the graft may show signs of diastolic dysfunction [8].

### 2.6. Evaluation of Tissue Doppler Imaging

Tissue Doppler imaging parameters are also altered in the normal cardiac graft, with e’ and s’ velocities lower than in normal population [26]. Both restrictive filling patterns and indirect signs of elevated filling pressures are often seen early post transplantation; they improve within the first post-transplant year and carry no prognostic value [27]. On the other hand, in some patients, restrictive physiology can be identified many years after HT, and this finding correlates with history of AR and heart failure episodes [28].

### 2.7. Evaluation of Postoperative Pericardial Effusion

Pericardial effusion has a high prevalence in HT patients as an early response to surgical injury or as the result of mismatch in volumes between the donor and the recipient heart. It may be seen in up to two-thirds of patients at 3 months but has a tendency to reduce over time [29,30]. However, a significant pericardial effusion may also be due to the effect of some immunosuppressive drugs, to an infective pericarditis in the immune-depressed patient, or to AR. Although these effusions rarely evolve into cardiac tamponade, frequent echocardiographic assessments are warranted, in order to detect extension and hemodynamic impact. Moreover, even the occurrence of hemodynamically irrelevant pericardial effusion is associated with an increased risk of hospitalization and mortality [31] and may in rare occasions lead to the development of constrictive pericarditis [32].

## 3. Primary Graft Dysfunction

Primary graft dysfunction (PGD) is the most common cause of early mortality after HT and occurs within 24 h after completion of transplant surgery. The ISHLT Consensus guidelines distinguish between PGD and secondary graft dysfunction, which may be attributed to a recognized cause, including pulmonary hypertension, hyperacute rejection, surgical complication and sepsis. In this setting, donor or recipient risk factors and intraprocedural aspects can further PGD. As a consequence of large discrepancies in treatment of left and right ventricular failure, PGD is further classified as left ventricular and right ventricular; left ventricular PGD also includes biventricular dysfunction and may be graded as mild, moderate and severe, depending on the necessity to adopt pharmacological inotropic support or mechanical circulatory support in order to maintain perfusion [33,34]. In this context, echocardiography has a role as a first-line diagnostic tool for detection of impaired left ventricular function (EF < 40%) and right ventricular function (i.e., TAPSE < 15) and exclusion of other causes of hemodynamic impairment. Frequent echocardiographic reassessments are crucial in the evaluation of graft function improvement in response to inotropic or mechanical circulatory support.

## 4. Echocardiography in Graft Rejection

The manifestation of rejection can occur from the intraoperative period to many years after transplant, and the timing of rejection has a significant role in establishing cause and diagnosis. Early graft dysfunction can be primary graft dysfunction, which does not include rejection etiology, or secondary graft dysfunction. In the latter condition, hyperacute graft rejection must be considered in case of either AB0 incompatibility or preformed cytotoxic antibodies that direct their activity against significant histocompatibility (MHC) antigens on the allograft. Late graft dysfunction includes AR, which can be cellular or antibody (humoral) mediated. Acute cellular rejection (ACR) is due to major and minor histocompatibility antigens, which are not equally expressed among all individuals; these proteins may act as alloantigens and activate alloimmunity by stimulating cytotoxic T cells [35]. Antibody-mediated humoral rejection (AMR) is poorly understood, but what is known is that the antibodies react with donor MHC antigens (HLA-I and HLA-II), leading to capillary endothelial changes with the deposition of immunoglobulin and complements within the myocardial capillary bed [36]. For a comprehensive description of AR, see Table 1. Notably, recent studies have established that effector B cells, and accordingly AMR, are involved in the development of CAV [37]. Other causes of allograft failure include recurrence of myocardial conditions such as amyloidosis, sarcoidosis, giant cell myocarditis, hereditary hemochromatosis, and malignancies like primary cardiac lymphoma.

Symptoms are uncommon in early stages of rejection, but eventually heart failure or sudden cardiac death can occur.

EMB is the gold standard for detection of graft rejection. However, the procedure carries the risk of complications, like cardiac tamponade, pulmonary embolism, pneumothorax, and damage to the tricuspid valve. Moreover, the patchy distribution of rejection may result in sampling errors, and interobserver variation in the interpretation of histological specimens may cause underestimation of the severity of rejection.

**Figure 4:**
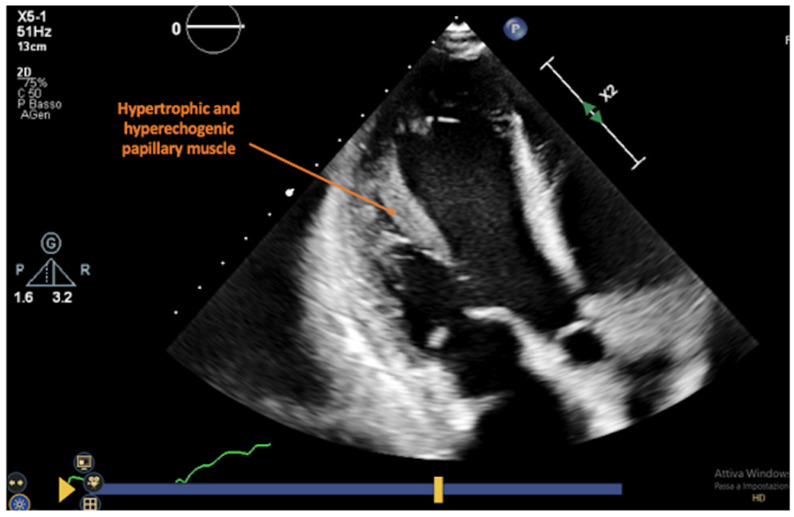
Four-chamber view focusing on the anterolateral papillary muscle that appears hypertrophic and hyperechogenic (ACR grade 3R at the endomyocardial biopsy).

During AR, conventional echocardiography may detect changes in myocardial structure (LV wall thickening and mass increase, changes in myocardial echogenicity) (Figure 4) and function (decrease in LV ejection fraction and/or abnormalities in regional wall motion), or the appearance of pericardial effusion [38,39]. These conventional echo parameters are usually late findings and indicate a higher grade of rejection, while on the other hand, they do not correlate with the severity of rejection detected by EMB [40].

Abnormalities in diastolic function appear earlier during graft rejection. They are caused by myocardial edema and interstitial fibrosis, which alter regional myocardial stiffness before affecting myocardial contractile function. Doppler indices of mitral inflow, including increased E/A ratio, shortening of isovolumic relaxation time and mitral valve pressure half time, have been the first and most extensively explored [41,42]. However, no single Doppler parameter or combination of parameters is powerful enough to detect AR. The main reasons are the strong dependency of Doppler-derived measurements on loading conditions and the difficulty in obtaining clear Doppler waves from transplant patients because of tachycardia and fusion of mitral E and A waves. Moreover, cardiac grafts frequently exhibit baseline diastolic filling abnormalities unrelated to AR and may gradually develop restrictive patterns beyond the first year of follow-up.

Since graft rejection affects both systolic and diastolic function, attempts have been made to investigate the myocardial performance index (MPI), a Doppler-derived combination of systolic and diastolic time intervals, as a possible early marker of acute rejection [43,44,45]. However, AR is associated with both an increase in isovolumic contraction time and decrease in isovolumic relaxation time, which explains the controversial results regarding the accuracy of MPI in detecting graft rejection.

Tissue Doppler imaging (TDI) enables the measurement of systolic and diastolic velocities within the myocardium, providing parameters that are not preload-dependent. Most studies have revealed a reduction in systolic and diastolic myocardial velocities during AR: Dandel et al. reported the association of peak systolic and early diastolic peak velocities obtained at basal posterior LV wall with AR [46]; Lunze et al. have identified a <15% decline in peak systolic (s’) and <5% decline in late diastolic velocity (a’) to individually predict non-rejection with 99% accuracy in a pediatric population [47]. Mankad et al. described that the sum of lateral mitral annulus systolic and diastolic velocities s’ and e’ > 13.5 cm/s determined by color-coded tissue Doppler had 93% sensibility, 78% specificity and 98% negative predictive value for predicting rejection grade 1B [48]. More recently, Ruiz Ortiz et al. confirmed these data and, among a wide set of echo parameters, reported that an s’ + e’ value ≥ 23 cm/s had a negative predictive value of 98% for ruling out rejection grade ≥ 2R [49]. However, myocardial velocities must be interpreted with caution in HT: translational allograft motion affects TDI parameters, inducing inter-patient variability in measurements; also, TDI velocities are low shortly after transplantation and gradually increase over the first year, remaining lower in transplanted hearts than in the general population [8]. Finally, the power of studies previously performed on TDI in AR is limited by small sample size, single-center analysis and lack of validation [50].

Nevertheless, constant TDI velocities during follow-up (change < 10% compared with baseline) show good accuracy in excluding, rather than predicting, AR [46]. Thus, the detection of a change in myocardial motion velocity during follow-up is more useful than the absolute value of one single measurement.

**Figure 5:**
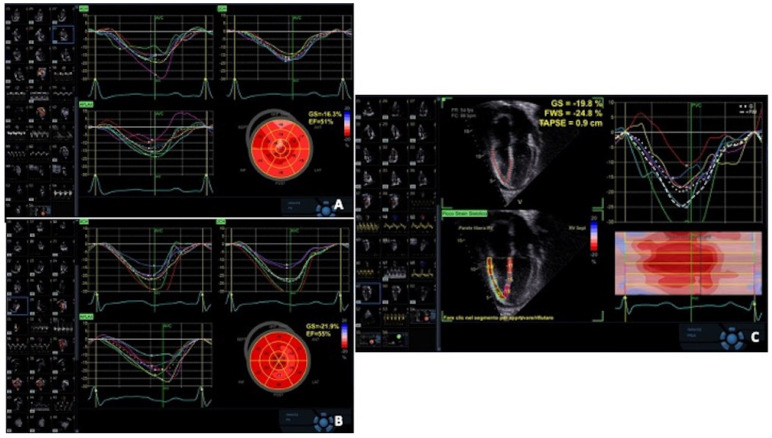
Global longitudinal strain (GLS) measured by speckle tracking echocardiography showing a reduced GLS for the left ventricle (3R—acute cellular rejection was confirmed by endomyocardial biopsy) (**A**); a normal GLS for the left ventricle (acute cellular rejection resolution confirmed by endomyocardial biopsy) (**B**); a reduced GLS for the right ventricle (3R—acute cellular rejection was confirmed by endomyocardial biopsy) (**C**).

Strain and strain rate echocardiography allow quantitative assessment of regional myocardial wall motion, reflecting both systolic and diastolic function, relatively independent of overall cardiac motion, which is more prominent in allografts. Myocardial deformation imaging has been shown to detect changes in regional systolic function at an earlier subclinical stage than conventional echocardiography. It can be derived either from TDI-based velocity measurements or from 2D STE. First findings on the usefulness of myocardial deformation imaging in early detection of subclinical rejection (grade 1B) were provided by TDI-derived data [51,52]. Interestingly, Marciniak et al. found that only regional strain from the lateral wall was predictive for acute rejection, not the regional strain from the septum, which is probably due to paradoxical septal motion that happens after cardiac surgery, including heart transplantation. However, an important limitation of TDI strain measurements is angle dependency. Two-dimensional STE overcomes this limitation and offers better spatial resolution, but requires good image quality [53]. Although different STE-derived parameters, including global longitudinal strain, radial strain and circumferential strain, have been investigated [54,55], most studies have described a significant correlation of LV global longitudinal strain (GLS) reduction with even mild acute rejection. Clemmensen et al. reported that GLS measured by STE was significantly reduced during moderate 2R-ACR and improved significantly in the resolving period, thus providing a dynamic monitor during treatment [56] (Figure 5A,B).

Mingo Santos et al. examined the RV free wall longitudinal strain in addition to LV strain parameters. An RV free wall longitudinal strain of less than 17% and an LV-GLS of less than 15.5% were independently associated with the presence of ACR of grade ≥ 2R, with negative predictive value of 98.8% in each case [57] (Figure 5C). Antonczyk et al. also investigated RV free wall strain and adopted a similar cut-off value < 16.8% for detection of ACR of grade ≥ 2R, with a negative predictive value of 95% [58]. On the other hand, other investigators found no correlation between ventricular strain and rejection, cellular or humoral [59,60]. Two recent meta-analyses, both concluding that GLS assessment of the LV may be useful in the detection of ACR [61,62], point out that the body of evidence on the diagnostic utility of GLS in ACR screening is largely based on observational studies. Therefore, heterogeneous results may be affected by differences in study design, lack of correspondence between different STE software packages and results, sampling errors in EMB and the fact that AMR has not been ruled out. STE detects subclinical graft dysfunction, irrespective of the cause. Notably, Ciarka et al. reported that patients with AMR showed a decline in GLS and global circumferential strain in the months preceding rejection (GLS < 15.5% and GCS < 15.2% could distinguish, with a sensitivity and specificity of 100.0%, AMR from controls 3 months before rejection) while control and ACR patients had stable strain values except for the moment of rejection [63].

Recently, STE has also been investigated in the context of multiparametric monitoring strategies: Clemmensen et al. developed a non-invasive model combining a change in LV-GLS and biomarkers for the detection of AR: a sudden drop in graft function, defined as a drop in LV-GLS ≥ −2% combined with either an increase in Troponin T ≥ 20% or NT-pro-BNP ≥ 30% compared with the levels at the last visit, showed a sensitivity of 49% and a specificity of 98% for the detection of ≥2R ACR [64].

Up to this point, no single echocardiographic parameter alone could be used for prediction of AR. However, as long as certain echo parameters (LV wall motion and myocardial strain, RV free wall strain) remain unchanged compared with the previous examination, the probability of rejection is very low, whereas, with the appearance of multiple predictors, the probability of rejection is significant.

Table 2 summarizes the literature on the diagnostic value of echocardiography in AR detection.

## 5. Echocardiography in CAV

CAV is a leading cause of late mortality and morbidity following HT, affecting almost 50% of patients within 5 years of cardiac transplant [65]. It is a diffuse, rapidly developing obliterative vasculopathy involving both large epicardial vessels and distal coronary microcirculation. The pathogenesis is complex and lies in the interplay between transplant-related factors (rejection episodes, especially antibody-mediated, cytomegalovirus infection and abrupt mode of donor death) and traditional cardiovascular risk factors (hypertension, hyperlipidemia, diabetes) [66,67]. Given the absence of afferent autonomic innervation, most HT recipients do not experience angina pectoris and may present with silent myocardial infarction, allograft dysfunction or sudden death [68]. ICA is recommended as the cornerstone technique for early CAV detection, while concurrent intravascular imaging using IVUS permits earlier detection of neointimal hyperplasia and has predictive value [2,69]. Table 3 describes CAV classifications using ICA.

However, ICA may underestimate the involvement of small distal vessels and overlook the occurrence of functional coronary alterations independent of morphological changes, while holding potential for procedure-related vascular injuries and contrast-related kidney damage.

CCTA has the potential, in experienced hands, for the early detection of coronary vessel wall changes, including atherosclerotic plaques and intimal hyperplasia, with the advantages of good spatial resolution [4,70]. Current guidelines recommend the use of CCTA as a non-invasive alternative for detection of CAV in >2 mm epicardial vessels (Class IIa, Level of evidence B recommendation) [2]. Still, the major concerns remain the exposure to ionizing radiation and nephrotoxic contrast. Renal impairment from multiple etiologies is common in HT patients, and contrast agents may precipitate kidney injury. Another method for CAV assessment is represented by single-photon emission computed tomography (SPECT) myocardial perfusion imaging (MPI). Studies of the diagnostic accuracy of SPECT MPI have reported variable sensitivity and specificity, and low to intermediate diagnostic accuracy in CAV detection. The main limitation in the diagnostic performance of SPECT is the diffuse nature of CAV disease, causing a scattered impairment in myocardial perfusion, with a lack of normal reference segments [2]. There remains a need to find a non-invasive kidney-friendly modality that can detect early development of CAV.

Standard two-dimensional echocardiography at rest has limited diagnostic accuracy for the detection of CAV. LVEF is usually within the normal range even in advanced forms of CAV, indicating the need for more-sophisticated non-invasive methods to detect impaired myocardial function caused by CAV. Nevertheless, late reduction of LVEF is often correlated with CAV and carries a poor prognosis [10,71]. The onset of new regional wall motion abnormalities should raise suspicion of CAV progression and prompt further investigation [72]. However, this is not a specific finding, as it may develop even in the absence of CAV or AR.

Diastolic dysfunction in CAV, related to the subversion of extracellular matrix by fibrosis and microvascular remodeling, develops prior to systolic impairment [73,74]. A restrictive filling pattern (defined as E/A velocity ratio > 2, IVRT < 60 msec, DT < 105 msec) is generally present in patients with severe CAV. Therefore, worsening of diastolic function during follow-up, although not specific for CAV, should prompt further evaluation [73]. Wall motion velocity analysis by PW-TDI appears to be suitable for the earlier detection of myocardial dysfunction in CAV: Dandel et al. found that reduced systolic radial wall motion peak velocity (Sm < 10 cm/s) in repeated measurements showed a sensitivity of nearly 90% for angiographic and/or IVUS detectable CAV in non-rejecting heart recipients, but the sensitivity decreased to 51% for detection of focal stenosis of major epicardial vessels [75]. Since endomyocardial fibers, which are mainly longitudinally oriented, are the most susceptible to macro- or microvascular ischemic insult, a reduction in GLS rest values has been associated with CAV in many reports [76,77]. Clemmensen et al. also reported that the entity of GLS reduction correlates not only with the presence but also with the severity of CAV, even in patients with preserved LVEF [76].

Stress echocardiography has been widely investigated as a non-invasive alternative imaging modality for the detection of CAV.

Exercise echocardiography is considered of limited value in HT patients because of resting tachycardia due to parasympathetic denervation, of impaired chronotropic response to exercise and of the diffuse nature of CAV abnormalities, which may result in balanced ischemia [78]. For this reason, current ISHLT guidelines no longer recommend exercise echocardiography for the detection of CAV [2]. Nevertheless, a recent report pointed out that exercise can induce a level of cardiac stress that is equal to or greater compared to dobutamine in HT patients who are able to exercise and prefer exercise stress testing [79].

Among pharmacologic stressors, dobutamine is the first choice because denervation of the transplanted heart increases the responsiveness to chronotropic stimulation [80], although some reports have described the adoption of dipyridamole in stress echocardiography for CAV surveillance [81].

Dobutamine stress echocardiography (DSE) has been the most widely used non-invasive tool to detect inducible ischemia in HT patients. Even so, the diagnostic value of DSE for detection of CAV remains unclear because of the wide range in reported sensitivity and specificity [82,83,84,85,86]. In a large meta-analysis by Elkaryoni et al., the sensitivity of DSE to detect CAV varied from 1.7% to 93.8% (pooled 60.2%), and specificity from 54.8% to 98.8% (pooled 85.7%) [87]. This variability may be explained with differences in the adopted definition of CAV, as DSE generally fails to detect mild CAV due to its diffuse nature; also, the accuracy of DSE depends on whether the gold standard adopted for comparison is ICA or IVUS [8,88]. Despite the suboptimal sensitivity for CAV, DSE still has important prognostic value: a positive dobutamine test, and even more, worsening of serial DSE, were found to be independent predictors of cardiac events and death during follow-up, while a normal DSE predicts an uneventful clinical course [89,90] and may justify postponement of invasive studies and CCTA in patients at high risk for AKI [2].

The accuracy of conventional stress echocardiography might be improved with the concurrent adoption of advanced echocardiographic techniques: quantitative analysis of segmental LV motion through strain rate imaging can increase DSE sensitivity in the detection of CAV from 63 to 88% [91]. Quantitative myocardial contrast echocardiography provides assessment of relative myocardial blood volume (rBV, a measure of microvascular density at rest), and its exchange after contrast bubble disruption induced by ultrasound could accurately detect severe CAV: an rBV < 14% at rest correlates with coronary intima thickness > 1 mm as determined by IVUS with a sensitivity of 90% and specificity of 75% [92]. However, these techniques require highly experienced professionals and advanced technologies that limit their widespread availability and application.

Coronary microvascular dysfunction defined by means of reduced coronary flow reserve (CFR) has emerged as a strong predictor of outcome in HT patients, also showing good accuracy for the detection of maximal intimal thickness of 0.5 mm on IVUS [93,94]. CFR is actually sensitive to both macrovascular and microvascular function and can be impaired before coronary abnormalities are even discernible on ICA. On this subject, Sade et al. reported that the assessment of CFR during DSE improved the sensibility and diagnostic accuracy of the latter method [95]. Otherwise, Pichel et al. proposed that the inclusion of CFR (with cut-off value < 2) during dipyridamole stress echocardiography could increase the negative predictive value for moderate-severe CAV [96].

Finally, Bjerre et al. have presented the combination of LV GLS and CFR as a feasible, reproducible and promising tool for non-invasive assessment of CAV and prognosis in HT patients: worsened LV GLS (>−15.5%) and low CFR (<2.0) were both independent predictors of major adverse cardiac events, while combined CFR and LV GLS represented a strong model to rule out significant CAV (CAV 2 and CAV 3) with NPV of 94.5% [97].

Table 4 summarizes the literature on the diagnostic value of echocardiography in CAV detection.

## 6. Echocardiography during Endomyocardial Biopsy

Traditionally, EMB has been performed with fluoroscopic guidance. Echocardiography is increasingly being adopted in this context because of several advantages: it avoids repeated X-ray exposure and may be performed at patients’ bedside when required. Echocardiographic monitoring permits adequately following the movement of the catheter in the RV and selecting the site of the biopsy, usually the apical segment of the right side of the interventricular septum [98,99]. It also affords the possibility of avoiding damage to the tricuspid valve, chordae and papillary muscles and promptly identifying the occurrence of complications like pericardial effusion [24,99,100]. In patients with difficult bioptome placement because of unusual anatomy or with a history of repeated biopsies of the same site (which degrade the ability to interpret the specimen for histologic evidence of rejection), the adoption of real-time 3D echocardiography may enhance the ability of the operator to identify the bioptome tip location within the RV [101].

## 7. Conclusions

Echocardiography is a primary non-invasive modality for the assessment of HT recipients. It is a versatile tool, providing information on both cardiac structure and function. It can be easily performed at bedside, serially repeated with no risk for the patient. For the detection of graft rejection, EMB is the gold standard and can not be replaced by standard echocardiography. Nevertheless, advanced echocardiographic techniques, like tissue Doppler imaging and strain imaging, in particular GLS, seem to be promising tools in the early detection of graft dysfunction [56,63]. In the detection of CAV, conventional stress echocardiography provides limited sensitivity, which may be improved by the adoption of speckle tracking techniques and CFR assessment. Nevertheless, stress echocardiography has recognized prognostic value in the assessment of CAV and represents an effective tool in the context of non-invasive multimodality imaging strategies, providing tailored screening modalities for patients that cannot afford to be investigated with contrast-bearing technologies.

## 8. Future Directions

Reliable and objective non-invasive modes of surveillance for HT recipients have to become relevant in clinical practice in order to reduce invasive and risky procedures.

Larger multicenter investigations and randomized controlled studies have to be performed to close the “gap in evidence” caused by small sample size, single center analysis and lack of validation of previously performed echo studies.

Actually, the overall reported sensitivity and specificity of single echo parameters are at most moderate, but multiparametric assessment and algorithm development have the potential to offer a predictive tool for cardiac graft complications.

## Figures and Tables

**Table 1:** Acute cardiac allograft rejection grading, both cellular and antibody-mediated according to ISHLT grading.

**ACUTE CELLULAR REJECTION**	**ISHLT-1990 ACR GRADE**	**ISHLT-1990 SEVERITY**	**HISTOPATHOLOGIC FINDINGS**	**ISHLT-2004 ACR GRADE**	**ISHLT-2004 SEVERITY**	**HISTOPATHOLOGIC FINDINGS**
	**0**	No ACR	No significant abnormality	**0R**	No ACR	No significant abnormality
	**1A**	Focal, mild ACR	Focal perivascular and/or interstitial infiltrate without myocyte damage	**1R**	Mild, low-grade ACR	Interstitial and/or perivascular infiltrate with up to one focus of myocyte damage
	**1B**	Diffuse, mild ACR	Diffuse infiltrate without myocyte damage			
	**2**	Focal, moderate ACR	One focus of infiltrate with associated myocyte damage			
	**3A**	Multifocal, moderate ACR	Multifocal infiltrate with myocyte damage	**2R**	Moderate, intermediate ACR	Two or more foci of infiltrate with associated myocyte damage
	**3B**	Diffuse, moderate ACR	Diffuse infiltrate with myocyte damage	**3R**	Severe, high-grade ACR	Diffuse infiltrate with multifocal myocyte damage +/− edema, +/− hemorrhage +/− vasculitis
	**4**	Severe ACR	Diffuse, polymorphous infiltrate with extensive myocyte damage +/− edema, +/− hemorrhage + vasculitis			
**ANTIBODY-MEDIATED** **REJECTION**	**ISHLT-2013 AMR GRADE**				**SEVERITY**	**HISTOPATHOLOGIC FINDINGS**
	**pAMR 0**				Negative for pathologic AMR	Histologic and immunopathologic studies are both absent
	**pAMR 1 (H+)**				Histopathologic AMR alone	Histologic findings are present: large endothelial nuclei, macrophage accumulation within vascular lumen, edema, necrosis, capillary fragmentation. Immunopathologic findings are absent.
	**pAMR 1 (I+)**				Immunopathologic AMR alone	Histologic findings are absent. Immunopathologic findings are present: C4d, CD68, CD3, pan-B-cell CD20, CD31/34, complement proteins
	**pAMR 2**				Pathologic AMR	Histologic and immunopathologic findings are both present
	**pAMR 3**				Severe pathologic AMR	Interstitial hemorrhage, capillary fragmentation, mixed inflammatory infiltrates, endothelial cell pyknosis, and/or karyorrhexis, and marked edema with immunopathologic findings are present

ACR = acute cellular rejection; AMR = antibody-mediated rejection; ISHLT = International Society of Heart and Lung Transplantation.

**Table 2:** Summary of literature on diagnostic value of echocardiography in AR detection.

Authors	Parameter	N Patients	EMB Rejection	Sensibility	Specificity	NPV
Paulsen et al. [38]	Increased LV mural thickness and mass Abnormal diastolic function	9	NA	NA	NA	NA
Ciliberto et al. [39]	Increased LV mural thickness and mass, reduction in LV EF, RV dilatation and wall motion impairment, pericardial effusion, increased myocardial echogenicity	21	NA	89 with 1 parameter 72 with ≥2	90 with 1 parameter 100 with ≥2	NA
Dandel et al. [40]	Sm of basal posterior wall ≥ 10% reduction Ea of basal posterior wall ≥ 10% reduction	190	Clinically relevant rejection *	88 89	95 97	97 98
Sun et al. [41]	≥2 among: Pericardial effusion, IVRT < 90 msec, Mitral inflow E/A ratio > 1.7	264	≥1B	57	54	68
Valantine et al. [42]	IVRT and PHT shortening E peak velocity increase	22	NA	NA	NA	NA
Vivekananthan et al. [43]	MPI increase ≥ 20%	20	≥3A	90	90	NA
Burgess et al. [44]	MPI	50	NA	NA	NA	NA
Bader et al. [45]	MPI	54	NA	NA	NA	NA
Dandel et al. [46]	Sm > 10% reduction Em > 10% reduction TEm > 10% extension	363 pt	Clinically relevant rejection *	88.33 91.66 93.33	94.06 92.08 95.05	93 94.8 96
Lunze et al. [47]	LV S’ −15% reduction LV a’ −5% reduction	122 pt	≥2R or AMR	AU ROC 0.93	NA	99
Mankad et al. [48]	s’ + e’ > 13.5 cm/s	78	≥1B	93	71	
Ruiz Ortiz et al. [49]	S’ + E’ > 23 cm/s	37	≥2R	NA	NA	99
Marciniak et al. [50]	LVPW Radial S ≤ 30% LVPW Radial SR < 3.0 s^−1^	31	≥1B	85 80	90 86	93 90
Kato et al. [52]	Systolic strain −27.4% Diastolic SR −2.8 s^−1^	35	≥1B	82.2 75.6	82.3 74.9	82.3 75
Sera et al. [54]	2D-STE-GLS < 14.8%	59	≥1B	64	63	
Sehgal et al. [55]	peak systolic longitudinal strain, radial strain circumferential strain	82	≥2R	Significant decline during rejection: *p* = 0.05 *p* = 0.03 *p* = 0.05		
Clemmensen et al. [56]	GLS	64	≥2R	Significant decline during rejection: −14.6% (−16.1 to −13 at baseline) vs. −13.3 (−14.9 to −11.8 at rejection). *p* = 0.0208		
Mingo-Santos et al. [57]	LV GLS < 15.5% RV FW < 17% LV + RV	34	≥2R	85.7 85.7 100	91.1 81.4 77	98.8 98.8 100
Antonczyk et al. [58]	4CH LS ≤ 13.8% RVFW ≤ 16.8%	45	≥2R	87 73	72 82	97 95
Ambardekar et al. [59]	GLS, GCS, CSSR, CDSR, LSSR, LDSR	30	NA	NS	NS	NS
da Costa et al. [60]	LV GLS, RV FW	54	≥2R	NS	NS	NS
Ciarka et al. [63]	GLS < 15.5% GCS < 15.2	403	AMR	100	100	

* Clinically relevant rejection = EMB grade 2 rejection or grades 1A and 1B accompanied by clinical symptoms; IVRT = isovolumic relaxation time, PHT = pressure half time; Sm = peak systolic wall motion velocity by PW-TDI from the posterior basal wall; Em = early diastolic wall motion velocity; TEm = early diastolic time (from onset of second heart sound to peak of Em) obtained by PW-TDI from the posterior basal wall; LV S’ = peak systolic TDI derived from the basal lateral LV wall; LV a’ = late diastolic velocity TDI derived from the basal lateral LV wall; s’ + e’ = sum of lateral mitral annulus systolic and diastolic velocities determined by color-coded tissue Doppler; S’ + E’ = sum of lateral mitral annulus systolic and diastolic velocities determined by pulsed tissue Doppler; 2D-STE-GLS = 2D STE derived global longitudinal strain; S = strain; SR = strain rate; LV GLS = left ventricle global longitudinal strain; RV FW = free wall right ventricular longitudinal strain; 4CH LS = four-chamber longitudinal strain; GLS = global longitudinal strain; GCS = global circumferential strain; CSSR = circumferential systolic strain rate; CDSR = circumferential diastolic strain rate; LSSR = longitudinal systolic strain rate; LDSR = longitudinal diastolic strain rate; AMR = antibody mediated rejection.

**Table 3:** ISHLT-recommended nomenclature for coronary allograft vasculopathy.

Classification	Severity	Angiographic Findings
**CAV 0**	Non-significant	No detectable angiographic lesion
**CAV 1**	Mild	Angiographic LM < 50% or Primary vessel with maximum lesion < 70% or Branch stenosis < 70%
**CAV 2**	Moderate	Angiographic LM < 50%, Single primary vessel ≥ 70% or Isolated branch stenosis in 2 systems ≥ 70%
**CAV 3**	Severe	Angiographic LM ≥ 50% or ≥2 primary vessels ≥ 70% or Isolated branch stenosis in all 3 systems ≥ 70% or CAV1 or CAV2 with allograft dysfunction (LVEF ≤ 45%) or evidence of significant restrictive physiology

CAV = coronary allograft vasculopathy; ISHLT = International Society of Heart and Lung Transplantation; LM = left main coronary artery; LVEF = left ventricular ejection fraction.

**Table 4:** Summary of literature on diagnostic value of echocardiography in CAV detection.

Authors	Parameter	Nr. Pts.	Sensitivity	Specificity	NPV
Barbir et al. [71]	LVEF < 60% with M-mode TTE	91	NA	NA	NA
Clemmensen et al. [76]	FS, TT and S’ GLS Decreased LVEF	198	92.7% 94.5%	42.0% 24.6%	71.6% 66.4%
Cohn et al. [78]	WMA at exercise-TTE, inducible ischemia, resting WMA	51	15%	NA	NA
Gebska et al. [79]	DSE vs. exercise TTE, LVEF	81	NA	NA	NA
Akosah et al. [80]	WMA at DSE	21	NA	NA	NA
Ciliberto et al. [81]	Resting Echo WMSI	21	poor	high	89%
Derumeaux et al. [82]	WMA at DSE	37	86%	91%	91%
Spes et al. [84]	WMA at DSE Systolic septum/posterior wall thickening at MMode in 2DDSE	28	79% 85%	83% 71%	91%
Chirakarnjanakorn et al. [85]	DSE	497	7%	98%	41%
Mahmoodurrahman et al. [86]	DSE and ICA	99	3.2 ± 3.3%	94 ± 2.9%	NA
Clerkin et al. [88]	DSE	154	0%	99%	81.7%
Bacal et al. [89]	WMA at DSE	39	64%	91.3%	84%
Spes et al. [90]	2D Resting Echo 2D DSE Serial rest Echo Serial DSE	109	57% 72% 60% 47%	88% 88% 71% 72%	51% 62% 80% 44%
Eroglu et al. [91]	IVS thickness, LV posterior wall and the LV EDD and ESD, LVEF, LV mass	42	63%	88%	92%
Rutz et al. [92]	rBV < 14%.	45	90%	75%	NA
Tona et al. [93]	Resting WMA CFR	73	57%	85%	85%
Tona et al. [94]	ED thickness of IVS and posterior wall, LVEF and CFR using CE-TTE	22	80%	100%	89%
Sade et al. [95]	CFR WMSI at DSE CFR and DSE	24	100% 55.6% 77.8%	64.3% 64.3% 87.2%	100% 69.2%
Pichel et al. [96]	Rest WMA CFR	74	15.3% 72.7%	96.7% 49.2%	84.2% 91.1%
Bjerre et al. [97]	LV-GLS and CFR	98	84.2%	67.5%	94.5%

DT = deceleration time; CAD = coronary artery disease; CFR = coronary flow velocity reserve; DSE = dobutamine stress echocardiography; LVEF = left ventricular ejection fraction; IVSd = interventricular septal thickness in end-diastole; LVEDD = left ventricular end diastolic diameter; LVESD = left ventricular end systolic dimension; LVIDd = left ventricular internal diameter in end-diastole; LVIDs = left ventricular internal diameter in end-systole; LVPWd = left ventricular posterior wall thickness in end-diastole; LV-GLS = left ventricular global longitudinal strain; WMA = wall motion anomalies; WMSI = wall motion score index; rBV = relative myocardial blood volume.
